# Next-generation mpox vaccines: efficacy of mRNA-1769 compared to modified vaccinia virus Ankara in non-human primates

**DOI:** 10.1038/s41392-024-02058-x

**Published:** 2024-11-20

**Authors:** Leonie Mayer, Leonie M. Weskamm, Marylyn M. Addo

**Affiliations:** 1 Institute for Infection Research and Vaccine Development (IIRVD), Center for Internal Medicine, University Medical Center Hamburg-Eppendorf, Hamburg, Germany; 2 Department for Clinical Immunology of Infectious Diseases, Bernhard Nocht Institute for Tropical Medicine, Hamburg, Germany; 3 German Center for Infection Research, Partner Site Hamburg-Lübeck-Borstel-Riems, Hamburg, Germany

**Keywords:** Preclinical research, Vaccines, Experimental models of disease, Infectious diseases

In a recent study published in *Cell*, Mucker et al. demonstrated in a non-human primate (NHP) model of lethal mpox virus (MPXV) infection, that a novel mRNA-based vaccine, as compared to modified vaccinia virus Ankara (MVA) strain 572, closely related to the standard-of-care MVA-BN vaccine, provides similar protective efficacy against lethality but superior efficacy against disease.^1^ In the mRNA-vaccinated group, a reduction in the number of lesions and viremia was associated with both neutralizing and Fc-mediated antibody functionality.

MPXV is a zoonotic Orthopoxvirus endemic in Western and Central Africa that causes mpox disease in humans. A clade II MPXV strain spread globally in 2022 and a more lethal clade Ib strain is causing substantial mortality in Central Africa since 2023. Both outbreaks were declared public health emergencies of international concern (PHEIC). MVA, historically licensed against smallpox, provides cross-protection against mpox and reduces disease in at-risk populations in real-world effectiveness studies.^2^ The development of several next-generation mpox vaccines is underway to improve large-scale production and enhance long-term efficacy.

**Fig. 1:**
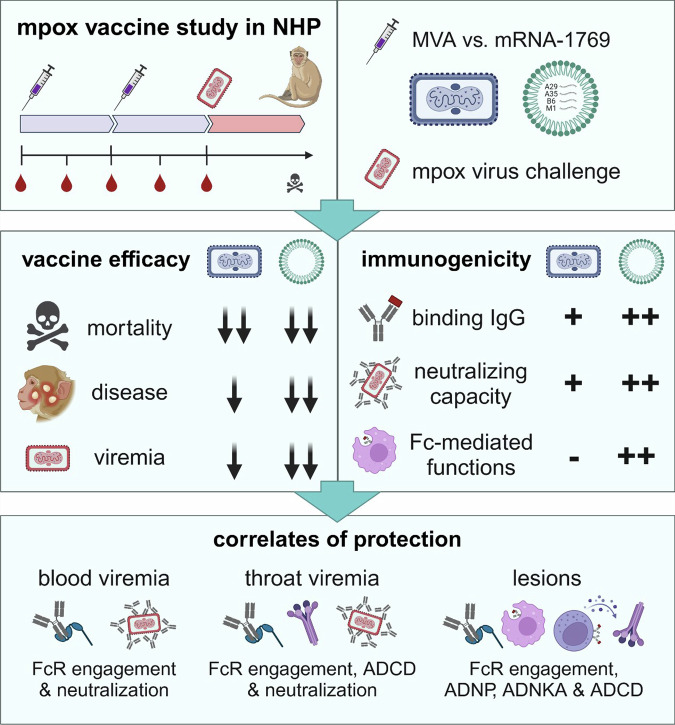
Study overview. As recently published in *Cell*, Mucker et al. conducted an mpox vaccine study in non-human primates (NHP), comparing a novel mRNA vaccine (mRNA-1769) to a live-attenuated Modified Vaccinia virus Ankara (MVA) vaccine. Following vaccination, NHPs were challenged with the mpox virus. While vaccination with mRNA-1769 and MVA resulted in similar protection against lethality, mRNA-1769 vaccination led to a stronger reduction of mpox lesions and viremia and induced superior antibody titers and functionality. A multivariate correlates analysis revealed that neutralizing and Fc-mediated antibody functions were associated with vaccine-induced protection. Created in BioRender

Mucker et al. tested the efficacy of an mRNA-lipid nanoparticle vaccine (mRNA-1769) in comparison to a live-attenuated MVA vaccine in an NHP model of lethal MPXV infection (Fig. 1). mRNA-1769 encodes highly conserved Orthopoxvirus antigens exposed on the surface of the mature virion (M1 and A29) and the enveloped virion (A35 and B6) with the rationale of generating immune responses against both infectious forms of the virus and increasing the likelihood of cross-protection.^3^ NHPs received two doses, 4 weeks apart, of either MVA (subcutaneously) or mRNA-1769 (intramuscularly) and were subsequently challenged intravenously with MPXV (clade 1 Zaire 1979 strain). Importantly, while 5/6 unvaccinated animals succumbed to disease, all vaccinated animals survived, showing the protective efficacy of MVA and mRNA-1769. However, the mRNA vaccine provided enhanced protection from disease, resulting in a reduction of blood and throat viremia, number of MPXV lesions, and disease duration.

To investigate if vaccine immunogenicity is associated with this differential protection from disease, the authors profiled the antibody responses in detail. While both vaccines elicited binding IgG antibodies against MVA lysate and variola virus antigens, the mRNA vaccine induced significantly higher titers against the M1, A29, A35, and B6 antigens. The authors also evaluated the neutralizing activity against a range of Orthopoxvirus strains, revealing a superior cross-neutralization capacity of serum from mRNA- compared to MVA-vaccinated animals. Using systems serology, Mucker et al. measured antibody isotypes, IgG subclasses, Fc receptor (FcR) engagement as well as Fc-mediated antibody functions such as antibody-dependent complement deposition (ADCD), phagocytosis by monocytes and neutrophils (ADCP, ADNP) and NK cell activation (ADNKA). These functions were shown to contribute to protection against several pathogens.^4^ In brief, Fc functionality was significantly higher in mRNA- compared to MVA-vaccinated animals. While each vaccinated animal showed a unique antibody profile, a principal component analysis revealed that animals clustered by the vaccine they had received, underlining the diverging immune responses elicited by MVA and mRNA-1769.

NHPs are a valuable model for evaluating novel vaccine candidates, as they mirror human disease and immunity more closely than mice. In addition, the possibility of viral challenge in NHPs allows to investigate correlates of protection. The authors identified several predictive antibody features associated with lower viral loads and lesion numbers in the mRNA-vaccinated group. Key predictors that contribute to the control of viral replication were virus neutralization as well as EV-specific FcR engagement and ADCD. In turn, EV-specific FcR engagement, ADNP and ADCD as well as MV-specific ADNP and ADNKA may be critical in limiting lesion formation. Assessing the persistence of memory B and T cell responses would be important for understanding if mRNA-1769 can provide long-term protection.

A major strength of the study by Mucker et al. is the inclusion of an MVA vaccine as a benchmark. This direct comparison of efficacy and immunogenicity with MVA puts the data into a clinically relevant context, eventhough the MVA used in this study (strain 572) is not identical with the licensed MVA-BN vaccine against mpox. Importantly, vaccination with mRNA-1769 reduced MPXV lesions and viral loads in the blood and mucosa of NHPs more efficiently than MVA. If transferable to humans, these two features may limit MPXV transmission. From a public health point of view, this would be highly relevant for controlling current and future MPXV outbreaks.

Interestingly, vaccination with MVA elicited lower antibody responses against the four antigens encoded by the mRNA vaccine. However, it still protected against lethality and has shown protective efficacy in humans. One factor that could explain the efficacy may be that MVA elicits a functional immune response against additional poxviral antigens that were not analyzed by Mucker et al. One explanation for the lower immunogenicity of MVA in this study may be the vaccine dosage.

Indeed, the selection of the vaccine dosages represents a limitation of the study. The authors compared a 150 µg dose of mRNA-1769, which is considerably higher than the licensed human dose of their mRNA-based COVID-19 vaccine (50–100 µg), to the licensed human dose of MVA (0.7 × 10^8^ PFU). On the one hand, they discuss that using a higher dosage of MVA could result in improved immunogenicity and efficacy against disease in NHPs and humans. However, manufacturing MVA dosages above 10^8^ PFU has been challenging. On the other hand, testing a range of mRNA-1769 dosages could have resulted in the identification of a lower dosage that provides protection but may be more transferable to human trials. Furthermore, alternative administration routes of the viral challenge might be explored in the future, as intravenous MPXV administration provides a stringent lethal model but might not reflect the natural route of infection in humans well.

A Phase I/II clinical trial (NCT05995275) is currently evaluating mRNA-1769 in humans. Another Phase I/II clinical trial (NCT05988203) is ongoing to test BNT166, another quadrivalent mRNA vaccine against mpox. Like mRNA-1769, BNT166 also encodes the A35, B6, and M1 antigens, but not A29, which is replaced by the MV antigen H3. BNT166 administered at a dosage of 30 µg showed protective efficacy in a lethal intratracheal MPXV challenge study in NHPs.^5^ These advances highlight the potential of mRNA vaccines as a promising tool against mpox, owing to their great advantages in terms of efficacy, immunogenicity, and flexibility in design and manufacture compared to traditional vaccine platforms.

In summary, the paper by Mucker et al. describes a well-designed challenge study in NHPs to test the efficacy of a novel mRNA-based vaccine compared to an MVA vaccine against mpox. The mRNA vaccine, as compared to MVA, conferred similar protection against lethality but superior protection against mpox disease. This study paves the road for further clinical development of this next-generation vaccine, and provides a better understanding of mpox correlates of protection, highlighting the role of Fc-mediated antibody functions.
