# Correction: Next-generation mpox vaccines: efficacy of mRNA-1769 compared to modified vaccinia virus Ankara in non-human primates

**DOI:** 10.1038/s41392-024-02092-9

**Published:** 2024-12-13

**Authors:** Leonie Mayer, Leonie M. Weskamm, Marylyn M. Addo

**Affiliations:** 1 Institute for Infection Research and Vaccine Development (IIRVD), Center for Internal Medicine, University Medical Center Hamburg-Eppendorf, Hamburg, Germany; 2 Department for Clinical Immunology of Infectious Diseases, Bernhard Nocht Institute for Tropical Medicine, Hamburg, Germany; 3 German Center for Infection Research, Partner Site Hamburg-Lübeck-Borstel-Riems, Hamburg, Germany

**Keywords:** Preclinical research, Vaccines, Experimental models of disease, Infectious diseases

Correction to: *Signal Transduction and Targeted Therapy* (2024) 9:327; 10.1038/s41392-024-02058-x; Article published online 20 November 2024

After the online publication of the research highlight,^1^ a reader pointed out that the MVA vaccine used in the original study by Mucker et al.^2^ was mistakenly labeled as “Jynneos”. The authors have now pointed out in the text, that the comparator vaccine used in the original study was the MVA-572 strain and not the MVA-BN (Jynneos) vaccine. The explanations “vaccine study” and “mpox virus challenge” were added to the first panel of Fig. 1 to clarify the study design. In the lower panel, the antibody functions were provided in more detail to avoid misunderstanding by the readers. The corrected figure is provided below. The main messages of this research highlight are not affected by these corrections.

Incorrect Figure
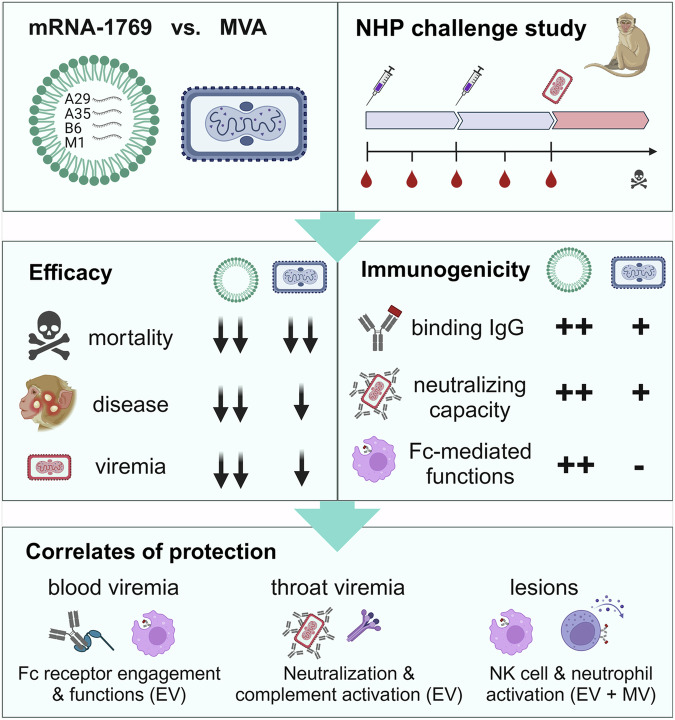


Correct Figure
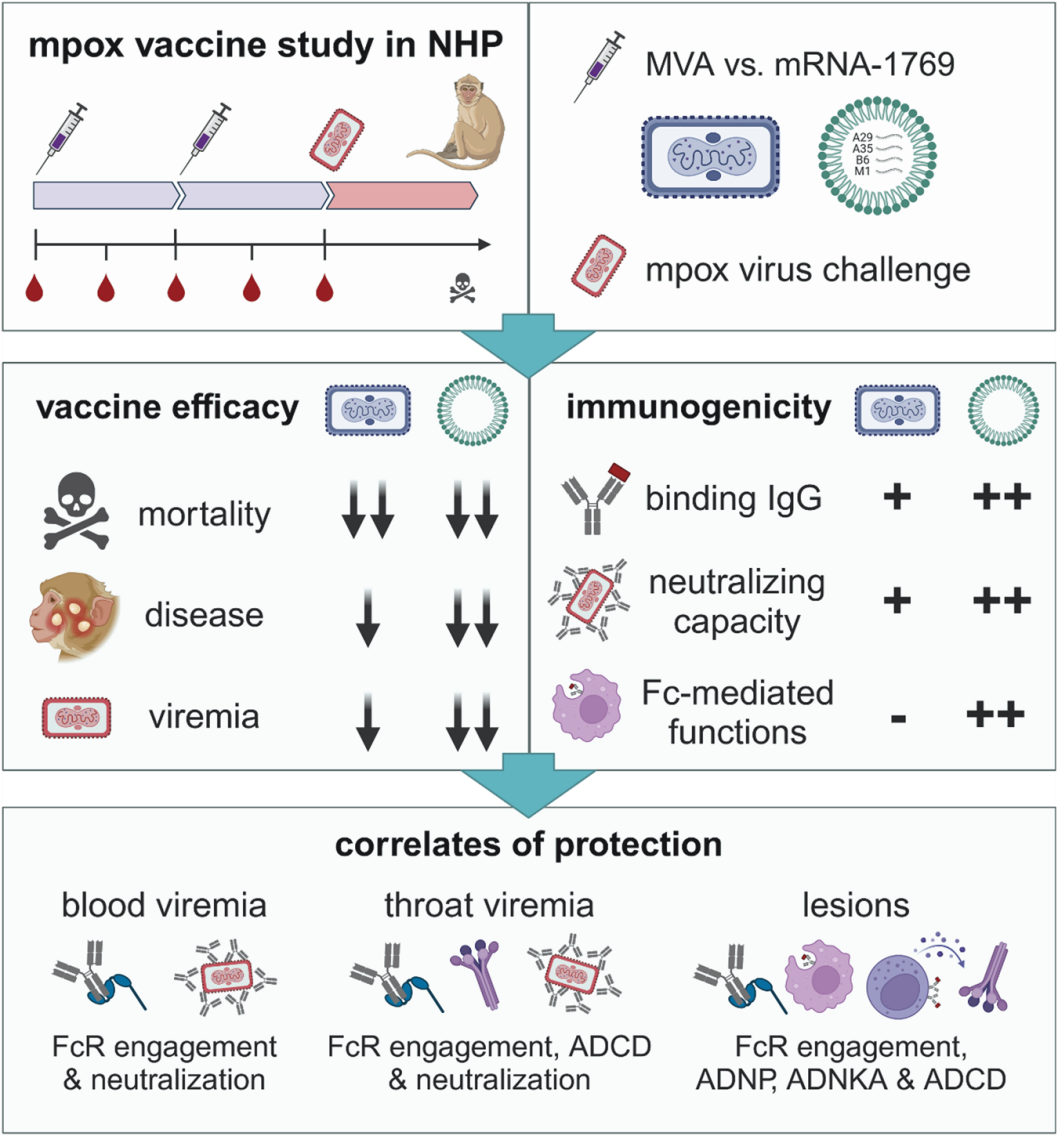


The original article has been corrected.
